# Efficacy of the Oral Administration of Maltodextrin Fructose Before Major Abdominal Surgery: A Prospective, Multicenter Clinical Study

**DOI:** 10.1007/s00268-022-06455-7

**Published:** 2022-06-19

**Authors:** Huanlong Qin, Jiafu Ji, Yi Miao, Tong Liu, Dongbing Zhao, Zhenyi Jia, Jun Jiang, Jiang Liu, Qiang Li, Xi Ji, Weihua Fu, Donghua Lou, Wenyu Xia, Ning Li

**Affiliations:** 1 Department of General Surgery, Shanghai Tenth People’s Hospital, Shanghai, China; 2 Department of Gastrointestinal Surgery, Peking University Cancer Hospital, Beijing, China; 3 Pancreas Center, Jiangsu Province Hospital, The first Affiliated Hospital of Nanjing Medical University, Nanjing, Jiangsu China; 4 Department of General Surgery, Tianjin Medical University General Hospital, Tianjin, China; 5 Department of Pancreatic and Gastric Surgical Oncology, National Cancer Center/National Clinical Research Center for Cancer/Cancer Hospital, Beijing, China; 6 Department of General Surgery, Shanghai Sixth People’s Hospital, Shanghai, China; 7 Department of General Surgery, Jinling Hospital, Medical School of Nanjing University, Nanjing, China; 8 Department of Anesthesiology, Shanghai Tenth People’s Hospital, Shanghai, China; 9 School of Public Health, Nanjing Medical University, Nanjing, Jiangsu China; 10 Department of Colorectal Disease, Shanghai Tenth People’s Hospital, Shanghai, China; 11 Jiangsu Chia Tai Fenghai Pharmaceutical Co. Ltd., Nanjing, Jiangsu China

## Abstract

**Background:**

To study the efficacy of the oral administration of maltodextrin and fructose before major abdominal surgery (MAS).

**Methods:**

This prospective, multicenter, parallel-controlled, double-blind study included patients aged 45–70 years who underwent elective gastrectomy, colorectal resection, or duodenopancreatectomy. The intervention group (IG) was given 800 mL and 400 mL of a maltodextrin and fructose beverage at 10 h and 2 h before MAS, respectively, and the control group (CG) received water under the same experimental conditions. The primary endpoint was insulin resistance index (IRI), and the secondary endpoints were fasting blood glucose, fasting insulin, insulin secretion index, insulin sensitivity index, intraoperative blood glucose, subjective comfort score, and clinical outcome indicators.

**Results:**

A total of 240 cases were screened, of which 231 cases were randomly divided into two groups: 114 in the IG and 117 in the CG. No time-treatment effect was detected for any endpoint. The IRI and fasting insulin were significantly lower in the IG than CG after MAS (*p* = 0.02 & *P* = 0.03). The scores for anxiety, appetite, and nausea were significantly lower in the IG than CG at 1 h before MAS. Compared with baseline, the scores for appetite and nausea decreased in the IG but increased in the CG.

**Conclusion:**

The oral administration of maltodextrin and fructose before MAS can improve preoperative subjective well-being and reduce postoperative insulin resistance without increasing the risk of gastrointestinal discomfort.

## Introduction

Insulin resistance (IR) is an important feature of the postoperative metabolic response. IR can decrease glucose uptake and utilization and increase endogenous glucose produced by gluconeogenesis in skeletal muscle and fat tissue [1, 2]. As a key process in the pathophysiology of surgical stress, perioperative IR can result in postoperative hyperglycemia, which has been associated with a 30% increase in the risk of postoperative infection [3]. Moreover, IR is linked to delayed recovery and increased incidence of mortality and major complications [4].

Perioperative oral carbohydrate (OCH) loading is a well-established strategy to reduce surgical stress and modulate insulin sensitivity during and after surgery. Our previous study showed that IR after colorectal cancer surgery was significantly higher in the fasting and placebo groups than in the OCH group [5]. A systematic review demonstrated that there was a significant reduction in IR following preoperative OCH loading. The maximum increase in the effect of insulin after carbohydrate ingestion on the morning of surgery was 50% [6].

Preoperative OCH loading is an important item in the Enhanced Recovery After Surgery (ERAS) protocol. A Cochrane review found that OCH significantly reduced time to flatus by 0.39 days (95% CI: 0.70–0.07) and length of hospital stay (LOS) by 0.30 days (95% CI: 0.56–0.04) compared with fasting or placebo [7]. Another meta-analysis showed that, in patients undergoing major abdominal surgery (MAS), preoperative OCH loading reduced the LOS by 1.08 days (95% CI: 1.87–0.29) [8]. OCH loading is recommended by several ERAS guidelines, including the ASA Committee, ERAS Society, and ESPEN [9–11].

The concept of preoperative OCH loading has been widely accepted across China in the past few years. The 2018 Consensus of ERAS jointly issued by the Surgical Branch and Anesthesiology Branch of the Chinese Medical Association recommends using this approach in clinical practice [11]. Some domestic carbohydrate preparations are commercially available in China. This multicenter study assessed the clinical effect of a carbohydrate mixture and provided evidence for the popularization and application of OCH loading.

## Methods

1. Study population: Patients were screened and enrolled from six hospitals in China (Shanghai Tenth People’s Hospital, Nanjing General Hospital of Nanjing Military Region, Beijing Cancer Hospital, Tianjin Medical University General Hospital, People’s Hospital of Jiangsu Province, and Cancer Hospital of the Chinese Academy of Medical Science) from September 2017 to October 2019. The inclusion criteria were (1) patients undergoing elective gastric surgery, colorectal surgery (CRS), or duodenopancreatectomy for the first time; (2) age 45–70 years; (3) signing of informed consent; (4) expected postoperative hospital stay of more than 72 h. The exclusion criteria were (1) patients with diabetes mellitus and other severe metabolic diseases; (2) patients undergoing emergency surgery or secondary surgery; (3) patients with inability to receive enteral nutrition; (4) patients with comorbidities to ensure the safety of the trial and minimize the occurrence of adverse events; (5) patients with mental disorders, alcohol addiction, or drug abuse history; (6) lactating and pregnant women; (7) patients with allergy to maltodextrin and fructose and various drugs; (8) participation in other clinical trials 3 months before the present study. The study was approved by the research ethics committee of each hospital and was registered in the Chinese Clinical Trial Registry. All subjects gave written informed consent.
2. Study groups: 240 patients were expected to enroll in this study, including 120 patients from the intervention group (IG) and 120 patients from the control group (CG). All patients fasted for 6 h before MAS. The IG was given 800 mL and 400 mL of a 12.5% maltodextrin/fructose mixture solution (Suqian, Jiangsu Chia Tai Fenghai Pharmaceutical Co., Ltd.) at 10 h and 2 h before operation, respectively, and the CG received the same volume of water under the same experimental conditions. All patients were received multimodal analgesia with temperature monitoring. The patients were encouraged to mobilize as soon as possible after operation. No glucose solution was infused intraoperatively in both groups. After surgery, glucose was administered at a dose of 2 g/kg of body weight at a flow rate of 60 drops per minute, and the daily volume was limited to 1500–2000 mL.
3. Study design: Randomization was stratified by research center. The study was parallel-controlled (allocation ratio of 1:1) and double-blind. The maltodextrin and fructose beverage bottle or water was assigned to patients using random numbers generated by SAS software version 9.3.1.
4. Primary endpoint: homeostasis model assessment insulin resistance index (HOMA-IRI) was calculated as fasting blood glucose level (mmol/L) × fasting insulin level (mIU/L)/22.5 and was measured before randomization (baseline) and on days 1 and 3 after MAS.
5. Secondary endpoints: (1) fasting blood glucose (mmol/L); (2) fasting insulin (μU/mL); (3) insulin secretion index (HOMA-β), calculated as 20 × fasting insulin level/(fasting blood glucose level—3.5); (4) insulin sensitivity index (HOMA-ISI), calculated as 1/(fasting blood glucose level × fasting insulin level). These four parameters were measured at baseline (before randomization) and on days 1 and 3 after MAS; (5) subjective well-being (anxiety, thirst, appetite, nausea, and fatigue) measured using a visual analog scale at baseline (before randomization) and 1 h before MAS. The following scoring system was used to assess the level of discomfort: 0, none; 1–3, mild; 4–6, moderate; 7–9, severe; 10, intense; (6) blood glucose measured at 30, 60, 120, and 180 min after the start of surgery; (7) clinical outcomes, including infectious and non-infectious complications, postoperative flatus time, hospitalization time, and incidence of pulmonary aspiration during anesthesia.
6. Data input and statistical analysis: One data administrator developed the data input system, and two administrators independently entered and reviewed data to ensure accuracy. Quantitative data were described using means and standard deviations. A two-way repeated measures ANOVA was performed to evaluate the effect of different interventions over time on primary and secondary endpoints mentioned above. For significant two-way interactions (time and interventions), we analyzed the effect of intervention on endpoints at every time point using ANOVA tests. Bonferroni correction for *p* value was used as appropriate. Dichotomous data were described as frequencies, and intergroup differences were analyzed using a *χ*2 test or Fisher exact test. Statistical analyses were performed using SAS software version 9.3.1. A two-tailed *p* value of less than 0.05 was considered significant.

## Results

**Fig. 1:**
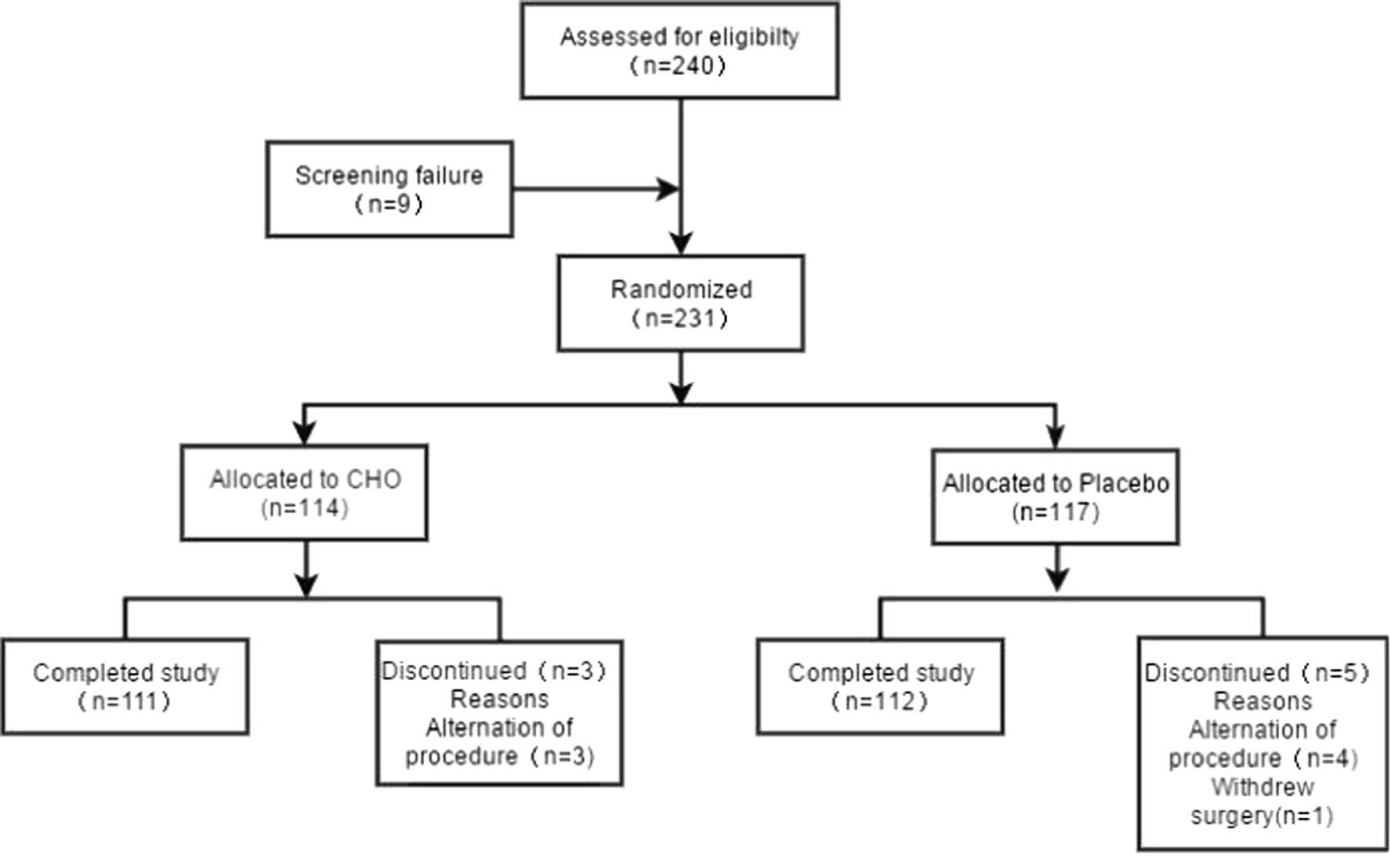
Flowchart of patient selection

1. Patient demographics and baseline characteristics: A total of 240 patients were screened, nine patients were excluded, and 231 patients were randomized (114 in the IG and 117 in the CG). Eight patients did not complete the study (Fig. 1) because of changes in surgical procedure (seven cases) or cancellation of surgery (one case). Demographics, comorbidities, type of surgery, and surgical status are shown in Table 1
2. HOMA-IRI: Table 2 showed the descriptive and test statistics of HOMA-IRI at each time point for both groups. Two-way repeated measures ANOVA results show non-significant interactions of time and intervention (*p* = 0.42), indicating the effects of interventions are not time dependent, that is, patients’ HOMA-IRI in both groups followed the same trend over time. On the other hand, there was a significant difference in HOMA-IRI between the IG and CG (group effect, *p* = 0.02), indicating that although the two groups showed similar trends over time, the CG showed significantly higher HOMA-IRI than the IG postoperatively. Subgroup analysis showed that there were significant group differences and time effects in HOMA-IRI in gastric and duodenopancreatectomy surgery patients. However, no significant differences were observed in terms of group effects within those who underwent colorectal and uncertain surgeries.
3. Secondary endpoints: Two-way repeated measures ANOVA results showed significant group effect (*p* = 0.01) and time effect (*p* < 0.001) but non-significant interactions of time and intervention (*p* = 0.65) in intraoperative blood glucose 30, 60, 120, and 180 min after the start of MAS (Table 3). Similarly, no significant time-effect interactions were detected for other secondary ending points, according to two-way repeated measures ANOVA (Table 4). Time effects are significant for fasting blood glucose, insulin secretion index, and insulin sensitivity index, while group effect is significant for fasting insulin only, indicating CG had significantly higher fasting insulin than the IG postoperatively (group effect, *p* = 0.03).
4. Subjective well-being: Group effects were significant in anxiety, appetite, and nausea, while time effect was only significant in thirst score. Specifically, the intervention group had significantly lower anxiety score both at baseline and preoperative period, but significantly higher appetite and nausea scores were only found at preoperative period in control group. Effects of interventions on subjective well-being scores were also not time-dependent, according to two-way repeated measures ANOVA results (Table 5).
5. Outcome indicators: The rate of infectious and non-infectious complications was non-significantly lower in the IG than in the CG (Table 6). Postoperative flatus time and hospitalization time were similar between the two groups (Table 7). There were no cases of pulmonary aspiration during anesthesia in our sample.

**Table 1:** Patient demographics and baseline characteristics

		Intervention group	Control group	*P* value
		(*n* = 114)	(*n* = 117)	
Age		58.39 ± 7.55	59.05 ± 7.65	0.66
Height (cm)		165.60 ± 7.40	164.00 ± 8.60	0.13
Weight (Kg)		63.10 ± 11.18	61.90 ± 10.73	0.41
Ethnicity	Han	112 (98.2%)	110 (94.0%)	0.10
	Others	2 (1.8%)	7 (6.0%)	
Gender	Male	73 (64.0%)	63 (53.8%)	0.12
	Female	41 (36.0%)	54 (46.2%)	
Bowel preparation	No	64 (56.1%)	62 (53.0%)	0.83
	Yes	47 (41.2%)	51 (43.6%)	
	Unknown	3 (2.6%)	4 (3.4%)	
Perioperative thermoregulation	No	61 (53.5%)	63 (53.8%)	0.96
	Yes	42 (36.8%)	44 (37.6%)	
	Unknown	11 (9.6%)	10 (8.5%)	
Anesthesia	General	112 (98.2%)	112 (95.7%)	0.72
	General + epidural	1 (0.9%)	3 (2.6%)	
	Unknown	1 (0.9%)	2 (1.7%)	
Type of surgery	Colorectal	31 (27.2%)	37 (31.6%)	0.70
	Gastric	55 (48.2%)	58 (49.6%)	
	Duodenopancreatectomy	19 (16.7%)	16 (13.7%)	
	Uncertain	9 (7.9%)	6 (5.1%)	
Surgical time (hours)		3.6 ± 1.17	3.5 ± 1.10	0.50
Preoperative waiting time (days)		1.50 ± 1.95	1.20 ± 0.87	0.13
Comorbidity	No	75 (65.8%)	75 (64.1%)	0.95
	Yes	30 (26.3%)	33 (28.2%)	
	Unknown	9 (7.9%)	9 (7.7%)	

Statistical analysis used: Differences of quantitative data between the two groups were compared using two-sample *t*-test. Differences of dichotomous data between the two groups were analyzed using *χ*2 test or Fisher exact test

**Table 2:** Descriptive and test statistics of insulin resistance index (primary endpoint) in the study groups (stratified by type of surgery)

		Descriptive statistics^a^				Two-way repeated measure ANOVA^b^		
Type of surgery	Group	*N*	Baseline	1 day after surgery	3 days after surgery	Group effect	Time effect	Group*time interaction
All surgery	Intervention	109	5.96 (9.49)	11.38 (16.11)	7.19 (7.3)	5.83 (0.02*)	4.58 (0.01*)	0.88 (0.42)
	Control	111	7.35 (17.14)	20.21 (42.27)	14.91 (59.48)			
Colorectal	Intervention	29	2.56 (4.97)	2.76 (2.31)	3.19 (3.38)	0.01 (0.91)	2.17 (0.12)	1.76 (0.17)
	Control	35	1.74 (2.52)	4.53 (7.73)	2.45 (2.55)			
Gastric	Intervention	55	5.65 (11.87)	13.78 (19.68)	7.14 (7.98)	6.24 (0.01*)	3.41 (0.03*)	0.82 (0.44)
	Control	59	8.87 (22.62)	30.01 (55.68)	22.55 (80.92)			
Duodenopancreatectomy	Intervention	18	12.24 (2.48)	16.32 (5.36)	12.98 (3.69)	5.83 (0.02*)	8.33 (< 0.001*)	0.10 (0.91)
	Control	15	14.77 (5.01)	18.61 (4.96)	14.59 (4.41)			
Uncertain	Intervention	7	6.27 (6.81)	15.56 (23.86)	9.32 (10.54)	0.01 (0.94)	1.12 (0.34)	0.02 (0.98)
	Control	2	4.96 (3.35)	17.84 (5.83)	9.95 (3.55)			

^a^The descriptive statistics are represented by mean (standard deviation)

^b^Test statistics for two-way repeated measure ANOVA are represented by *F* statistics (*p* value). The *p* value is interpreted as the probability against the hypothesis of no statistical differences across different intervention/time groups or intervention-time interaction as appropriate

*P < 0.05 as significant

**Table 3:** Descriptive and test statistics of intraoperative blood glucose (mmol/L) in the study groups (stratified by type of surgery)

		Time (min) after the start of surgery							Two-way repeated measure ANOVA^b^		
	Group	*N*	Baseline (before surgery)	30 min	60 min	120 min	180 min	Immediately after surgery	Group effect	Time effect	Group*time interaction
Descriptive statistics^a^	Intervention	87	5.42 (1.61)	6.12 (2.12)	6.57 (2.06)	6.78 (2.26)	6.98 (2.13)	7.25 (2.16)	6.49 (0.01)*	24.46 (< 0.001)*	0.66 (0.65)
	Control	86	5.41 (1.86)	6.28 (1.68)	6.74 (1.97)	7.37 (2.26)	7.57 (2.31)	7.69 (1.93)			

^a^The descriptive statistics are represented by mean (standard deviation)

^b^Test statistics for two-way repeated measure ANOVA are represented by *F* statistics (*p* value). The *p* value is interpreted as the probability against the hypothesis of no statistical differences across different intervention/time groups or intervention-time interaction as appropriate

*P < 0.05 as significant

**Table 4:** Descriptive and test statistics of other secondary endpoints in the study groups

		Descriptive statistics^a^				Two-way repeated measure ANOVA^b^		
Endpoint	Group	*N*	Baseline before surgery	1 day after surgery	3 days after surgery	Group effect	Time effect	Group*time interaction
Fasting blood glucose (mmol/L)	Intervention	110	5.34 (1.47)	7.26 (2.77)	6.10 (1.98)	0.56 (0.45)	51.38 (< 0.001*)	0.88 (0.42)
	Control	112	5.31 (1.66)	7.73 (3.40)	6.07 (1.88)			
Insulin secretion index	Intervention	110	1.36 (0.84)	2.68 (2.43)	1.83 (1.23)	1.19 (0.28)	43.82 (< 0.001*)	1.41 (0.25)
	Control	112	1.37 (1.25)	3.17 (2.89)	1.79 (1.24)			
Insulin sensitivity index	Intervention	110	1.00 (0.78)	0.59 (0.42)	1.04 (2.07)	0.12 (0.73)	6.04 (0.003*)	0.57 (0.57)
	Control	112	0.98 (0.63)	0.68 (0.99)	0.88 (1.47)			
Fasting insulin (μU/mL)	Intervention	109	24.44 (37.29)	34.40 (49.86)	26.31 (27.42)	5.07 (0.03*)	2.19 (0.11)	0.48 (0.61)
	Control	111	29.93 (75.83)	52.21 (96.5)	45.67 (139.63)			

^a^The descriptive statistics are represented by mean (standard deviation)

^b^Test statistics for two-way repeated measure ANOVA are represented by *F* statistics (*p* value). The *p* value is interpreted as the probability against the hypothesis of no statistical differences across different intervention/time groups or intervention-time interaction as appropriate

*P < 0.05 as significant

**Table 5:** Descriptive and test statistics of subjective well-being in the study groups

		Descriptive statistics^a^			Two-way repeated measure ANOVA^b^		
Subjective well-being index	Group	*N*	Baseline^c^	Preoperative^c^	Group effect	Time effect	Group*time interaction
Anxiety	Intervention	114	1.83 (1.86)	1.76 (1.71)	9.46 (0.002*)	0.02 (0.88)	0.04 (0.84)
	Control	116	2.43 (2.48)	2.43 (2.44)			
Thirst	Intervention	110	1.36 (0.74)	2.50 (1.85)	0.03 (0.86)	61.45 (< 0.001*)	0.25 (0.62)
	Control	113	1.41 (0.95)	2.41 (1.84)			
Appetite	Intervention	114	1.62 (1.77)	2.04 (2.03)	4.79 (0.03*)	0.29 (0.59)	2.48 (0.12)
	Control	117	2.35 (2.53)	2.15 (1.89)			
Nausea	Intervention	114	1.79 (1.47)	1.42 (1.67)	5.36 (0.02*)	0.03 (0.87)	3.19 (0.08)
	Control	117	1.89 (1.80)	2.19 (2.81)			
Fatigue	Intervention	111	1.18 (0.56)	2.62 (2.34)	1.20 (0.27)	86.99 (0.52)	0.17 (0.68)
	Control	113	1.08 (0.27)	2.40 (1.99)			

^a^The descriptive statistics are represented by mean (standard deviation)

^b^Test statistics for two-way repeated measure ANOVA are represented by *F* statistics (*p *value). The *p *value is interpreted as the probability against the hypothesis of no statistical differences across different intervention/time groups or intervention-time interaction as appropriate

^c^Baseline refers to subjective well-being before randomization; preoperative refers to subjective well-being at 1 h before surgery

^*^P < 0.05 as significant

**Table 6:** Surgical complications in the study groups

Type of complications	Group	No	Yes	Unknown	*P* value
Infectious	Carbohydrate-treated (*n* = 114)	98 (86.0%)	2 (1.8%)	14 (12.3%)	0.368
	Placebo (*n* = 117)	98 (83.8%)	6 (5.1%)	13 (11.1%)	
Non-infectious	Carbohydrate-treated (*n* = 114)	91 (79.8%)	9 (7.9%)	14 (12.3%)	0.959
	Placebo (*n* = 117)	95 (81.2%)	9 (7.7%)	13 (11.1%)	

Statistical analysis used: Differences between the two groups were analyzed using *χ*2 test

**Table 7:** Length of hospital stay and flatus time in the study groups

Index	Group	Time (days)	*P* value
Hospitalization time	Carbohydrate-treated (*n* = 114)	11.29 ± 6.56	0.6762
	Placebo (*n* = 116)	11.67 ± 7.20	
Flatus time	Carbohydrate-treated (*n* = 18)	3.29 ± 1.13	0.7902
	Placebo (*n* = 15)	3.25 ± 0.88	

Statistical analysis used: Differences between the two groups were analyzed using paired-*t*-tests

## Discussion

The oral administration of 800 mL and 200 mL of a carbohydrate solution at 10 and 2 h before MAS conformed to ERAS protocols. Evening doses increase hepatic glycogen storage, and a subsequent morning dose changes patient status from fasted to fed, potentially reducing IR [12]. Previous studies showed that the level of compliance with this protocol was high among CRS patients [13, 14]. In this study, all participants completed this protocol, proving its feasibility in clinical practice.

The carbohydrate concentration should be adjusted to maximize its effect on IR. In this respect, low-concentration glucose solutions, including 5% dextrose and 6–7% carbohydrate, do not have a significant metabolic effect by failing to stimulate insulin release. The dose used in this study—50 g of complex carbohydrates in 400 mL of fluid (equivalent to 12.5% carbohydrate)—decreases insulin resistance by 50% and has adequate osmolality for gastric emptying [14].

As reported in other studies, HOMA-IRI and fasting insulin were significantly lower in the IG than in the CG postoperatively, suggesting that preoperative OCH loading might increase insulin sensitivity, allowing reducing the amount of insulin to control blood sugar after surgery. However, there was no detectable effect of OCH loading on fasting blood glucose, HOMA-β and HOMA-ISI in the postoperative period. Subgroup analysis indicated that the effect of OCH loading differed among types of surgeries. In gastric and duodenopancreatectomy surgery patients, the alteration of HOMA-IRI was similar to that in all surgery. However, OCH loading had no detectable group or time effects on HOMA-IRI in patients who underwent CRS and uncertain surgery. The variable effect of OCH loading may be associated with the surgical site and degree of surgical stress. However, no time-treatment effect was detected in any outcome measurement in our study.

A major concern to anesthetists is the ingestion of 400 mL of a carbohydrate drink in the immediate preoperative period and the perceived elevated risk of pulmonary aspiration. A scintigraphic study employing a gamma camera showed that the gastric emptying of 400 mL of a 12.5% carbohydrate drink was achieved within 90 min in preoperative patients and healthy volunteers. Before anesthetic induction, there was no significant difference in residual gastric volumes between an intervention group receiving a 12.5% carbohydrate beverage and the placebo group [15]. Moreover, Kaska et al. showed that preoperative OCH loading did not reduce gastric emptying compared with fasting [16]. Our results corroborated the safety of this treatment because there were no cases of pulmonary aspiration during anesthesia in our sample.

Several studies reported that carbohydrate beverages improved perioperative well-being. Hausel et al. found that the IG was less hungry and less anxious than both the fasting and placebo groups in the presurgical period [17]. A meta-analysis showed that a carbohydrate beverage significantly reduced thirst, appetite, anxiety, and discomfort compared with fasting and placebo (flavored water) [6]. In contrast, the Cochrane Review found no significant difference in patient well-being postoperatively between the intervention and fasting groups [18]. Our previous study showed that OCH loading reduced appetite compared with fasting 1 h before surgery. In this cohort, the effect of carbohydrate was similar to that reported previously. Furthermore, OCH loading attenuated nausea preoperatively.

The effects of carbohydrate relative to placebo on clinical endpoints, such as postoperative complications and LOS, remain controversial. A randomized controlled trial on MAS found that hospital stay was shorter in the carbohydrate group [15]. A meta-analysis of 21 randomized clinical trials showed that preoperative carbohydrate treatment significantly reduced the length of hospital stay by 1.08 days (95% CI: 1.87–0.29) in patients undergoing MAS [19]. In contrast, a network meta-analysis did not support this conclusion, and OCH loading before elective surgery slightly decreased the length of postoperative hospital stay compared with fasting and had no benefit over water or placebo. Moreover, the rates of postoperative complications or secondary outcomes were similar between the intervention and placebo groups [7]. A PROCY study reported that preoperative OCH loading maintained blood glucose levels to < 180 mg/dL but did not reduce the risk of postoperative infectious complications compared with placebo in elective MAS (relative risk, 1.019; 95% confidence interval, 0.720–1.442, *P* = 1.00) [20]. In this study, we focused on MAS and, although the infection rate was lower and the LOS was shorter in the IG than in the CG, these differences were not significant. This result may be because ERAS measures have been used more often in the past few years and reduce the effect of OCH on postoperative clinical outcomes.

This study has several limitations. First, only patients aged 45–70 years with a BMI of 16.7–31.6 were enrolled, and those with severe metabolic diseases were not included. Second, the study is multicenter, and tests were performed in six biochemical laboratories, which might affect the consistency of the results. Third, the non-standardization of the evening meal consumed the day before the study might affect our data. Fourth, the null-significance in subgroup analysis could also subject to lower power due to the small sample sizes.

In conclusion, the oral administration of maltodextrin and fructose before MAS reduced IR in the postoperative period and improved subjective comfort preoperatively. Moreover, this treatment did not cause pulmonary aspiration during anesthesia, demonstrating its safety in clinical practice.
